# A Novel Pro-Melanogenic Effect of Standardized Dry Olive Leaf Extract on Primary Human Melanocytes from Lightly Pigmented and Moderately Pigmented Skin

**DOI:** 10.3390/ph14030252

**Published:** 2021-03-11

**Authors:** Shilpi Goenka, Sanford R. Simon

**Affiliations:** 1 Department of Biomedical Engineering, Stony Brook University, Stony Brook, NY 11794-5281, USA; sanford.simon@stonybrook.edu; 2 Department of Biochemistry and Cellular Biology, Stony Brook University, Stony Brook, NY 11794-5281, USA; 3 Department of Pathology, Stony Brook University, Stony Brook, NY 11794-5281, USA

**Keywords:** dry olive leaf extract, extracellular melanin, dendricity, tyrosinase, pro-pigmentation, lightly pigmented cells, moderately pigmented cells

## Abstract

Benolea^®^ (EFLA^®^943) is a standardized dry olive leaf extract (DOLE) considered safe for food consumption and has demonstrated superior pharmaceutical benefits such as antioxidant, anti-obesity, and anti-hypertensive activities. However, there is no study on its effects on melanogenesis yet. Disruption in the sequence of steps in melanogenesis can lead to hypopigmentary disorders which occur due to reduced production or export of pigment melanin in the skin. There is a need for safe and nontoxic therapeutics for the treatment of hypopigmentation disorders. Herein, we studied the effects of DOLE over a concentration range of 10–200 µg/mL on melanin synthesis and melanin secretion in B16F10 mouse melanoma cells and MNT-1 human melanoma cells and validated our results in primary human melanocytes (obtained from lightly pigmented (LP) and moderately pigmented (MP) cells) as well as their cocultures with keratinocytes. The capacity of melanocytes to export melanosomes was also estimated indirectly by the quantitation of melanocyte dendrite lengths and numbers. Our results show that DOLE significantly enhanced levels of extracellular melanin in the absence of effects on intracellular melanin, demonstrating that this plant extract’s pro-melanogenic activity is primarily based on its capacity to augment melanin secretion and stimulate melanocyte dendricity. In summary, our preliminary results demonstrate that DOLE may hold promise as a pro-pigmenting agent for vitiligo therapy and gray hair treatment by its exclusive and novel mechanism of functioning as a dendrite elongator. Further studies to elucidate the mechanisms of action of the pro-melanogenic activity and effects of DOLE on melanosome export as well as the last steps of melanogenesis are warranted.

## 1. Introduction

Skin pigmentation disorders, including vitiligo, post-inflammatory hypopigmentation, and progressive macular hypomelanosis, are the leading cause of psychosocial distress and impact the quality of life [1]. Around 0.5–2% of the world population is afflicted with vitiligo [2], while a higher rate of 8.8% has been reported in India [3]. Deficiency in the production and/or export of the pigment melanin causes hypopigmentation which generates an uneven skin tone marked by white-colored patches. In particular, the disorder of progressive macular hypomelanosis is characterized by impairments in the maturation of melanosomes and their export [4]. Hypomelanosis disorders that are hereditary comprise oculocutaneous albinism, Griscelli syndrome, Hermansky–Pudlak syndrome, and Chediak–Higashi syndrome [5,6]. Idiopathic guttate hypomelanosis (IGH) is another hypomelanosis disorder characterized by white macules [7]. The skin lesions of IGH patients were shown to have reduced melanin content or complete absence of melanin in keratinocytes with disrupted dendrite branching of melanocytes, indicative of a dysfunction in the normal export of melanin pigment from melanocytes to keratinocytes [8]. In the hair follicle, reduced melanin pigmentation manifests as graying of hair associated with declining antioxidant activity linked to the aging process [9]. Melanin is a polymeric pigment that is responsible for skin and hair coloration and is synthesized by melanocytes within their specialized vesicles called melanosomes [10]. The process of melanogenesis involves a multi-step pathway that utilizes the conversion of L-tyrosine into L-dihydroxyphenylalanine (L-DOPA) and its subsequent conversion to dopaquinone which is catalyzed by the rate-limiting enzyme tyrosinase [11]. In melanocytes, after synthesis and maturation through four distinct stages, melanosomes rapidly move along the microtubules while the movement of the mature melanosomes to the tip of the dendrite is achieved along actin filaments [12], after which the melanosomes are transferred and eventually phagocytosed by keratinocytes, thereby protecting them from UV-induced damage [13]. In the epidermis, the melanocytes and keratinocytes form a symbiotic unit; a single melanocyte supplies melanin pigment to up to 40 keratinocytes through its extensive network of dendrites [14]. Although the details of all the steps involved in the transfer of melanin from melanocytes and deposition to keratinocytes are still not fully understood [15,16], the visual appearance of melanin in the skin and the hair is due to its accumulation in keratinocytes via a process which incorporates melanocyte–keratinocyte interactions mediated by melanocyte extensions known as dendrites.

Current therapeutics for vitiligo comprise the use of topical corticosteroids, surgical treatments, topical immunomodulators, and narrow-band UVB phototherapy [17], which are used as monotherapy or combined with other modalities [18,19]. Despite their benefits, they have limitations that include reappearance of white lesions after the treatment is discontinued and other side effects, thus reducing patient compliance [20,21]. Medicinal plant-based extracts and compounds that can stimulate melanogenesis are in great demand for correcting the hypopigmentation of white skin patches in vitiligo; there is also a need in the cosmeceutical industry to achieve a more “natural” self-tanning effect, or for gray hair repigmentation. Moreover, enhanced melanogenesis may also help provide increased photoprotection and may reduce the risk of skin cancer. For example, chaste tree extract, a natural plant-based extract, has shown enhancement of pigment synthesis in melanocytes and has been incorporated into a commercial product [22]. Another study showed that piper nigrum extract showed a better capacity to stimulate melanocyte proliferation and pigmentation in human subjects as compared to its purified key alkaloid piperine [23].

Benolea^®^ (EFLA^®^943) is a patented dry olive leaf extract (DOLE) considered safe for food consumption and has demonstrated safety in preclinical studies where it was shown to possess anti-hypertensive effects [24,25,26,27] and improved cardiovascular complications by lowering blood pressure; its anti-hypertensive effects were comparable to that of a known drug, captopril [28]. DOLE was shown to protect human whole blood cells from DNA damage by enhancing the antioxidant capacity of these cells and scavenging radicals [29]. In another study, DOLE showed anti-inflammatory effects and protective effects from DNA damage on human arterial endothelial cells [30]. In addition, DOLE was shown to demonstrate protective effects in a liver injury model in rats [31,32] and exerted a potent neuroprotective effect on the hippocampus in a neuronal damage model in rodents in another study [33]. The safety and tolerability of DOLE for oral consumption at doses up to 500 mg twice daily have been established [28]. In addition, DOLE shows comparable antioxidant activity to vitamin C which is attributable to the polyphenol content of the extract. Due to the several protective properties of the 80% ethanolic extract of olive leaves, it has also been included in the European Pharmacopeia (Ph. Eur.) [34].

OLE has been shown to possess skin-whitening capacity based on previously published studies [35,36]. An olive extract (Cayoma^®^ Olive, Qenax AG, Switzerland) containing 12% olive polyphenols was shown to possess skin-lightening capacity [35]. Another study tested the efficacy of orally administered OLE (containing 15% oleuropein) in a UVB-irradiated mice model and demonstrated a reduction in the number of melanin granules in the skin tissue [36]. To the best of our knowledge, studies on the effects of DOLE on melanocytes in cell culture are lacking. Hence, in this study, we tested the effects of DOLE on melanogenesis in vitro using mouse and human melanoma cells and further validated the effects in normal human melanocytes from two different skin phototypes, representative of Asian and Caucasian skin types. Our results show a novel effect of DOLE which indicates its potential as a pro-melanogenic candidate instead of anti-melanogenic.

## 2. Results

### 2.1. Effects of DOLE on Melanogenesis in B16F10 Cells

**Figure 1:**
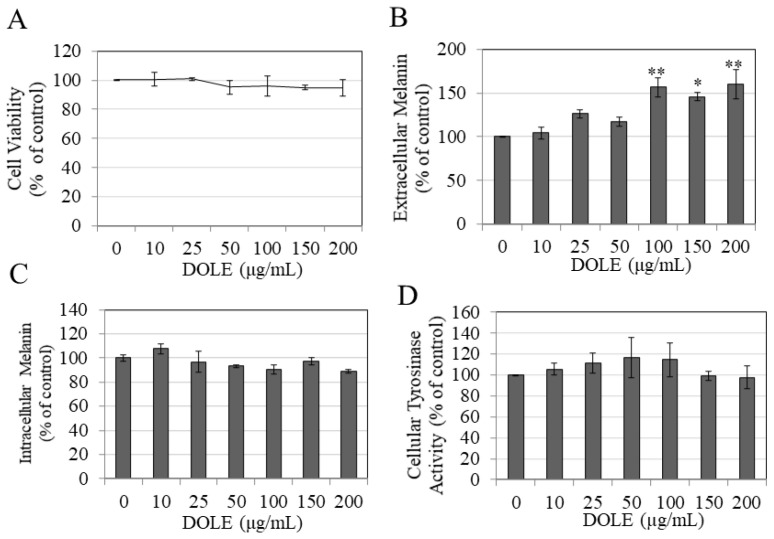
(**A**) Viability of B16F10 mouse melanoma cells treated with dry olive leaf extract (DOLE) over a concentration range (0–200 µg/mL) for a period of 48 h; data are mean ± SEM of three independent experiments. (**B**) Extracellular melanin and (**C**) intracellular melanin levels in B16F10 cell cultures treated for 48 h with different concentrations of DOLE; (**D**) effects of DOLE on cellular tyrosinase activity in B16F10 cells quantitated as melanin formation from L-DOPA; data for (**B**–**D**) are mean ± SEM of at least two independent experiments; * *p* < 0.05 vs. control, ** *p* < 0.01 vs. control; one-way ANOVA with Dunnett’s post hoc test.

As DOLE was nontoxic to B16F10 cells over the concentration range 10–200 μg/mL during the 48-h period (Figure 1A), these concentrations were selected for subsequent studies on melanin synthesis and export. Our results show that DOLE enhanced the secretion of melanin in the culture medium; significant increases of 56.70%, 45.53%, and 59.70% were obtained for DOLE concentrations of 100, 150, and 200 μg/mL, respectively (Figure 1B). The levels of intracellular melanin were unchanged for cells treated with DOLE at all concentrations (Figure 1C). Collectively, these results indicate that DOLE promoted the export of melanin from the cells into the medium. As tyrosinase is the primary rate-limiting enzyme in the pathway regulating melanogenesis, we next studied if the stimulation of melanin secretion by DOLE in B16F10 cells occurred via an increase in the activity of the enzyme tyrosinase. Our results show that DOLE did not have any effect on tyrosinase activity in the lysates of B16F10 cells at any concentration (Figure 1D).

### 2.2. Effects of DOLE on Melanogenesis in MNT-1 Cells

**Figure 2:**
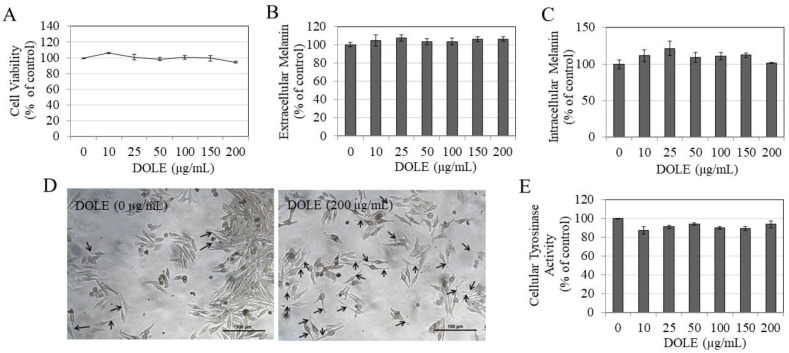
(**A**) Viability of MNT-1 human melanoma cells treated with different concentrations of DOLE for 48 h; data are mean ± SEM of three independent experiments. (**B**) Extracellular melanin and (**C**) intracellular melanin levels in cultures of MNT-1 cells treated for 48 h with different concentrations of DOLE; data for (**B**,**C**) are mean ± SEM of values from one representative experiment out of two experiments. (**D**) Representative images of MNT-1 cells for untreated and DOLE-treated groups, where elongated dendrites are shown by black arrows; (**E**) cellular tyrosinase activity in lysates of MNT-1 cells; data are mean ± SEM of three independent experiments; one-way ANOVA with Dunnett’s post hoc test.

DOLE showed no cytotoxicity to MNT-1 cells over the concentration range 10–200 μg/mL during the 48-h period (Figure 2A); hence, these concentrations were subsequently used for further testing. Our results show that while DOLE did not enhance the secretion of melanin in the culture medium of MNT-1 cells (Figure 2B) or alter the levels of melanin in MNT-1 cells at any concentration (Figure 2C), the microscopic observations revealed enhanced dendrite lengths in MNT-1 cells treated with DOLE at the higher concentration of 200 µg/mL, confirming the promotion of melanocyte activation by DOLE (Figure 2D). Our results of tyrosinase activity in lysates of MNT-1 cells treated with DOLE show no change at any concentration (Figure 2E). Collectively, the results indicate that DOLE promoted dendrite extension which is a necessary prerequisite for the export of melanin from the cells into keratinocytes.

### 2.3. Effects of DOLE on Melanogenesis in HEMn-LP Cells

**Figure 3:**
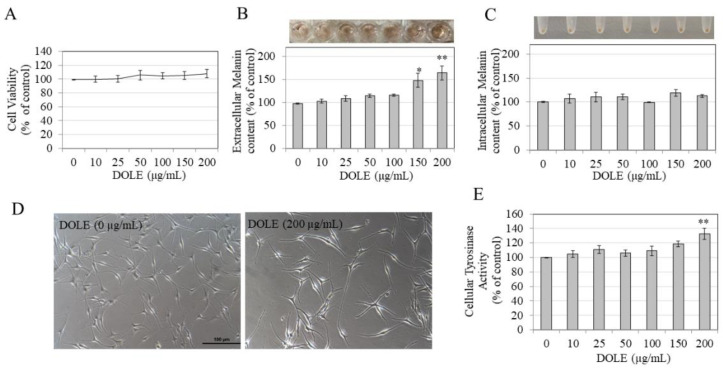
(**A**) HEMn-LP cell viability in the presence of different concentrations of DOLE; (**B**) extracellular melanin levels and (**C**) intracellular melanin levels in cultures of HEMn-LP treated for 48 h with different concentrations of DOLE; (**D**) representative phase-contrast micrographs showing cells treated without and with DOLE (200 µg/mL); (**E**) cellular tyrosinase activity in HEMn-LP cells after treatment with DOLE for 48 h. * *p* < 0.05 vs. control; ** *p* < 0.01 vs. control; one-way ANOVA with Dunnett’s post hoc test. Data for (**B**) and (**C**) are mean ± SEM of two independent experiments, while all other data are mean ± SEM of at least three independent experiments.

We next evaluated if our results of enhanced melanin export obtained earlier using B16F10 and MNT-1 melanoma cells might be retained in normal melanocytes derived from the human foreskin. We first tested DOLE on HEMn-LP cells, which are derived from Caucasian skin. Our results show that DOLE was nontoxic to HEMn-LP cells over the concentration range 10–200 μg/mL during the 48-h duration (Figure 3A); hence, these concentration ranges were used in subsequent experiments. We observed a strikingly darker culture medium for cells treated with DOLE at higher concentrations as compared to the control, indicative of melanin secretion into the medium for these cells (panel, Figure 3B). The measurements of extracellular melanin confirmed our visual observations; DOLE significantly increased extracellular melanin by 49.97% and 66.3% at 150 and 200 µg/mL, respectively (Figure 3B). We did not note any effect of DOLE at any concentration on the levels of intracellular melanin after visual inspection (panel, Figure 3C) as well as after spectrophotometric measurement (Figure 3C). As DOLE at 200 µg/mL showed the greatest response on extracellular melanin, we also evaluated the cellular morphology of cells to identify if these results correlated with the cellular events of increased dendricity which are needed for melanin transfer. Morphological changes induced by DOLE treatment were evident within LP cells, with markedly longer dendrites as compared to the control group (Figure 3D). To further assess the effects of DOLE on events associated with melanosome export, we also measured melanocyte dendrite lengths and numbers, which represent cellular events underlying the export of melanosomes.

Quantitation of dendricity indices showed that treatment of LP cells with DOLE at 200 µg/mL significantly stimulated the total dendrite length by 30.97%, without affecting the number of dendrites (Table 1). The average dendrite length was enhanced by 18.15% although levels were not statistically significant.

Our results further show that DOLE induced a significant stimulation of tyrosinase activity by 32.33% in HEMn-LP cells at the concentration of 200 µg/mL, while concentrations lower than 200 µg/mL did not affect intracellular tyrosinase activity (Figure 3E). Overall, these results indicate that in HEMn-LP cells, DOLE at 200 µg/mL exhibits a robust capacity to stimulate melanin export by increasing dendricity and export of melanosomes in the medium, which could be correlated with stimulatory effects on intracellular tyrosinase activity.

### 2.4. Effects of DOLE on Melanogenesis in HEMn-MP Cells

**Figure 4:**
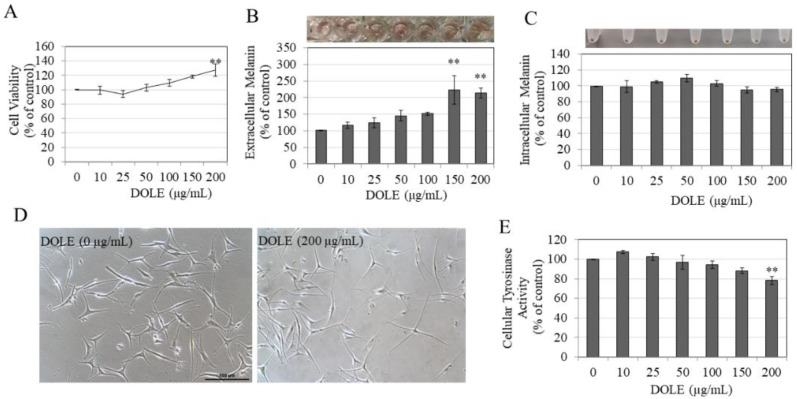
(**A**) HEMn-MP cell viability in the presence of different concentrations of DOLE for a duration of 48 h; (**B**) extracellular melanin and (**C**) intracellular melanin levels in cultures of HEMn-MP treated for 48 h with different concentrations of DOLE; (**D**) representative phase-contrast micrographs showing cells of control and cells treated without and with DOLE (200 µg/mL); ** *p* < 0.01 vs. control; one-way ANOVA with Dunnett’s post hoc test. (**E**) Cellular tyrosinase activity in HEMn-MP cells after treatment with DOLE for 48 h. All data are mean ± SEM of three independent experiments.

DOLE was next evaluated in HEMn-MP cells which are derived from Asian skin. Our results show that, similar to HEMn-LP cells, DOLE was nontoxic in HEMn-MP cells over the concentration range 0–200 µg/mL, although DOLE at 200 µg/mL significantly increased cell viability by 27.25% (Figure 4A). Similar to our earlier results of LP cells, we observed a darker-colored culture medium for cells treated with DOLE at higher concentrations, indicative of melanin secretion into the medium for these cells (panel, Figure 4B). The measurements of extracellular melanin revealed that DOLE induced a dose-dependent enhancement of extracellular melanin; a significantly higher increase of 121.65% and 112.37% was achieved by DOLE treatment at concentrations of 150 and 200 µg/mL, respectively (Figure 4B). The intracellular melanin levels were not altered in MP cells treated with DOLE at any concentration, which was confirmed visually (panel, Figure 4C) as well as by quantitation (Figure 4C).

The photomicrographs of cells treated with DOLE at 200 µg/mL showed cells with markedly longer dendrites as compared to the control group (Figure 4D). This was further confirmed by measuring dendricity indices in these cells, and our results are summarized in Table 2. DOLE significantly stimulated the total dendrite length of MP cells by 72.72% and dendrite number by 29 % (Table 2). The average dendrite length was also significantly enhanced by 29.04%.

DOLE showed a significant suppression of tyrosinase activity by 21.54% in HEMn-MP cells at the concentration of 200 µg/mL, while concentrations lower than that did not affect intracellular tyrosinase activity (Figure 4E). Interestingly, our results of intracellular tyrosinase activity in HEMn-MP cells show a marked difference from that of HEMn-LP cells in which DOLE stimulated tyrosinase activity at the same concentration.

### 2.5. Effects of DOLE on Melanogenesis in Cocultures of HEMn-LP and HEMn-MP Cells with Keratinocytes

**Figure 5:**
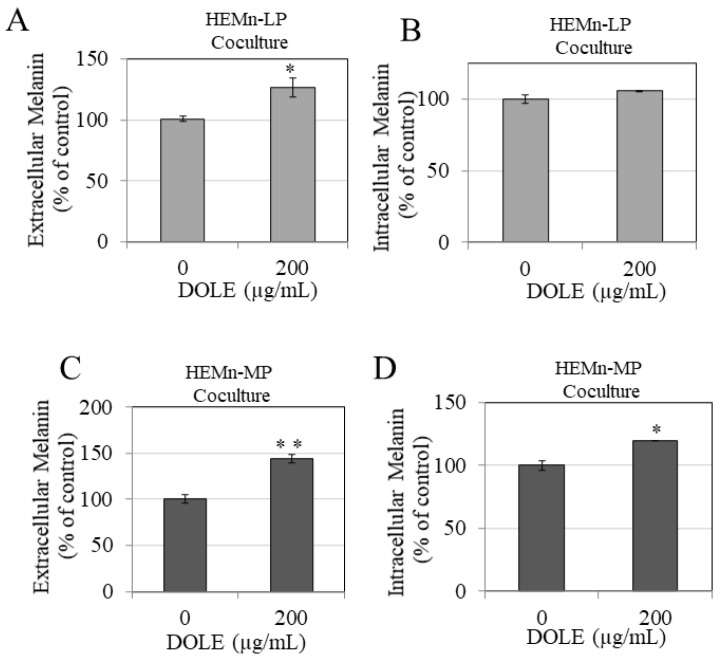
(**A**) Extracellular melanin and (**B**) intracellular melanin levels in cocultures of HEMn-LP cells treated in the absence or presence of 200 µg/mL DOLE; (**C**) extracellular melanin and (**D**) intracellular melanin levels in cocultures of HEMn-MP cells treated in the absence or presence of 200 µg/mL DOLE; all data are mean ± SEM of triplicates; * *p* < 0.05 and ** *p* < 0.01 vs. control unpaired student’s *t*-test.

Our results show that DOLE at 200 µg/mL significantly increased extracellular melanin in HEMn-LP cocultures with keratinocytes by 25.4% (Figure 5A), while the levels of intracellular melanin were unaltered (Figure 5B). Next, we examined the effects of DOLE in HEMn-MP cocultures with keratinocytes, and our results show that DOLE (200 µg/mL) showed a significant enhancement of extracellular melanin by 43.74% (Figure 5C). Interestingly, DOLE also enhanced the levels of intracellular melanin by 19.39% (Figure 5D).

Collectively, our results demonstrate the novel finding of the pro-melanogenic capacity of DOLE which has not been reported before. In addition, our results show that the pro-melanogenic capacity of DOLE is higher in melanocytes from Asian skin as compared to the Caucasian skin phototype.

## 3. Discussion

The advantages of better stability, ease of handling, and constituent homogeneity of dried plant extracts that are standardized to a specific bioactive have been an impetus for the use of such extracts instead of raw herbal drugs in the pharmaceutical industry [37]. Moreover, several previous studies have demonstrated the beneficial effects of DOLE, which, along with its aqueous solubility, provides an attractive candidate for further evaluation for the treatment of skin pigmentary disorders. A previous clinical study [38] showed that the application of a patented OLE-containing cream (SuperHeal™ O-Live Cream, PhytoCeuticals, Inc., Elmwood Park, NJ, USA) for two months led to facial rejuvenation; the authors attributed the effects to the photoprotective, antioxidant, and anti-inflammatory properties of OLE in the formulation. Although the melanin index was not changed from the baseline group, the authors further reported that 53% of the 36 study participants showed visual reduction in hyperpigmented facial spots. Since the formulation also consisted of ceramide in addition to OLE, the authors speculated that ceramide might have contributed to the effects observed. Nevertheless, studies to evaluate the effects of DOLE on melanocytes are still lacking, and it is currently unclear whether OLE can suppress or promote pigmentation. To the best of our knowledge, ours is the first study to report the effects of DOLE on mouse and human melanoma cells as well as primary melanocytes from two different donors, and we demonstrate a novel finding of selective augmentation of melanosome secretion.

Our study provides a proof of principle for the use of DOLE as a candidate medicinal plant extract for the treatment of hypopigmentation disorders of the skin. We conducted initial studies using the B16F10 mouse melanoma cell model which has been well validated for studies of compounds which can both modulate melanin levels within the cells (intracellular) and its secretion outside the cells (extracellular). We also assayed for the effects of DOLE using MNT-1 human melanoma cells which are more physiological as compared to mouse melanoma cells. Our results in MNT-1 cells show that DOLE seemed to promote the export of melanosomes, in the absence of changes in the levels of melanin within the cells or the intracellular tyrosinase activity, which is similar to our results obtained earlier with B16F10 mouse melanoma cells. In contrast to B16F10 cells, MNT-1 cells are known to be less sensitive to changes in extracellular melanin levels in the culture medium; rather, they exhibit changes in dendricity which is a cellular event linked to melanosome export from cells. However, to definitely establish the novel activity of DOLE, we also extended our experiments to primary cultures of normal human melanocytes, utilizing skin cells from donors which represent the Asian and Caucasian skin phototypes. This was further validated using a contact coculture system using these two melanocyte subsets with human keratinocytes. Although primary melanocytes do not release sufficient melanin into the culture medium as compared to melanoma cells, we noted that the medium was visually darker after treatment with DOLE at higher concentrations; our measurements of extracellular melanin by the spectrophotometric method confirmed that melanin granules were secreted out, in the absence of toxicity. Compounds which induce cytotoxicity to primary melanocytes can also cause an increase in extracellular melanin, but DOLE was nontoxic at the doses at which it stimulated extracellular melanin. Our results also show that the efficacy of DOLE in its capacity to stimulate melanin secretion was higher in HEMn-MP cells as compared to HEMn-LP cells, which might be related, at least in part, to the presence of the higher melanin content of MP cells but might also be due to difference in cell densities used in our experiments. Although it was apparent that DOLE seemed to have a much more pronounced effect on extracellular melanin levels and melanocyte dendrites in the case of HEMn-MP cells in contrast to HEMn-LP cells, especially at the concentration of 200 µg/mL, a statistical comparison is fitting for future investigation where equal cell numbers for both melanocytes as well as melanocytes from multiple donors are used.

Compounds which can target later steps in the melanin synthesis pathway to stimulate pigmentation are desirable. Increased pigmentation is attributed to a higher transfer of melanosomes synthesized within melanocytes to neighboring keratinocytes via dendrites which are cytosolic extensions of melanocytes and are the primary conduits of pigment transfer. Hence, the elongation of dendrite length and/or an increase in the number of dendrites can promote pigment transfer effectively to distant keratinocytes. In this study, we have established that DOLE is an exclusive stimulator of dendrite length, which explains, at least in part, the finding of augmented export of melanosomes in MP and LP cultures. This finding has not been observed before and presents a novel route by which DOLE enhances melanin transport by dendrite elongation, without affecting melanin levels within cells. A similar result has been reported before with piperine derivatives [39] which showed a similar result as that of DOLE with stimulation of dendrite lengths in the absence of changes in melanin synthesis, although piperine compounds also increased the dendrite number. Interestingly, DOLE did not affect the number of dendrites in LP cells, but selectively increased dendrite lengths; this might point to mechanisms which regulate dendrite extension. For example, the role of actin assembly in dendrite extension has been implicated previously [40,41] and the involvement of Rac1 and RhoA, members of the rho-subfamily of guanosine triphosphate (GTP)-binding proteins, in the regulation of dendricity has also been documented [42,43]. The mechanism of dendrite elongation was not studied in the present study and warrants further research.

The effects of DOLE on melanogenesis were also evaluated using a coculture model consisting of melanocytes in direct contact with keratinocytes mimicking the in vivo conditions where keratinocytes modulate melanogenesis by cellular cross-talk [44,45]. Interestingly, DOLE was shown to enhance intracellular melanin in HEMn-MP cocultures in contrast to HEMn-MP monocultures, which is indicative of a role for keratinocytes in modulating the effects of DOLE on melanogenesis. Although we did not note a similar occurrence in cocultures of HEMn-LP cells, it is possible that the presence of higher melanin levels (as in HEMn-MP cells) might have a contribution. The coculture model employed by us has several limitations. The ratio of melanocytes to keratinocytes in the epidermis is around 1:40 [14], while the contact coculture model employed by us consisted of melanocytes and keratinocytes at a ratio of 1:2 which is considerably lower than physiological conditions. However, maintaining a coculture of melanocytes and keratinocytes at close to physiological ratios without compromising cellular viability and melanin detection sensitivity is challenging. In addition, we did not evaluate if DOLE can in fact stimulate melanosome export by enhancing the uptake of melanosomes transferred from melanocytes to keratinocytes. Although we noted stimulation of extracellular melanin in cocultures of melanocytes as well, the levels were lower than those obtained when melanocytes were treated with DOLE at 200 µg/mL in monocultures. It is thus possible that part of the melanin exocytosed from melanocytes might have been exported into keratinocytes, although we did not evaluate for the presence of melanin granules within keratinocytes per se; hence, whether DOLE indeed stimulated the uptake of melanosomes into keratinocytes directly or via other mechanisms remains to be determined. We also evaluated DOLE over the duration tested in melanogenesis studies for any cytotoxicity to dermal fibroblasts, one of the other key cells surrounding melanocytes in the dermal junction; DOLE did not show any toxicity at all the tested concentrations (Figure S1B), establishing its safety for dermatological use. Although we selected a concentration range of 10–200 µg/mL in the present study, we also evaluated the cytotoxicity range for DOLE in LP and MP cells; our results show that DOLE was nontoxic up to concentrations of 1 mg/mL, while viability was diminished at 2 mg/mL in both LP and MP cells (Figure S2A,B).

Previous reports [33,46] have demonstrated the strong antioxidative potential of olive leaf extracts, and a recent study further documented that OLE that was standardized to oleuropein levels showed synergistic effects with UV filters and exerted improved photoprotective benefits, contributing to the in vitro sunscreen protection factor (SPF) of sunscreen formulations [47]. This can be beneficial for vitiligo therapeutics where the antioxidant activity in conjunction with pro-melanogenic activity may be desirable, given the role of oxidative stress in the pathophysiology of vitiligo. A previous study on piper nigrum extract revealed that the extract showed higher potency than the purified bioactive piperine in its capacity to stimulate repigmentation in vitiligo [23]. The complex interactions due to the presence of various other compounds in the total extract as opposed to the individual bioactive might lead to differences in potencies. Although we did not further examine which bioactive component in DOLE might be the key cause of the effects obtained in this study, we speculate that oleuropein, the primary phenolic bioactive in this extract, might be the primary ingredient responsible for the pro-melanogenic effects observed in this study. Further studies to test the effects of oleuropein on melanogenesis are currently ongoing.

## 4. Materials and Methods

### 4.1. Materials

Bicinchoninic acid (BCA) protein assay kit was procured from ThermoFisher Scientific. Cell lysis buffer was procured from Signosis Inc. (Santa Clara, CA, USA).

### 4.2. Plant Material

DOLE (Benolea^®^ (EFLA^®^943)) was obtained as a gift from Frutarom Switzerland Ltd. (Wadenswil, Switzerland). DOLE (lot# 4987563) standardized to 16–24% oleuropein and ≥30% polyphenols was obtained from dried leaves of the plant species *Olea europaea L.* by extraction using ethanol (80% m/m), followed by a patented filtration procedure (EFLA^®^ HyperPure) and subsequent drying to yield a brown powder. The stability and microbiological purity were confirmed by the manufacturer. The quantitative analysis of the constituents of DOLE by high-performance liquid chromatography (HPLC) has been reported previously [48], and they were shown to primarily consist of the phenolic oleuropein (17%), along with remaining compounds that include flavonoids, apigenine-7-O-glucoside, quercetin, and luteolin-7-O-glucoside (0.29%), tannins (0.52%), and caffeic acid (0.02%). The stock solution of the compound was prepared in sterile PBS and stored at −20 °C until use.

### 4.3. Cell Culture

B16F10 mouse melanoma cells were obtained from the American Type Culture Collection (ATCC; Manassas, VA, USA) and were cultured in Dulbecco’s modified Eagle medium (DMEM) supplemented with 10% heat-inactivated fetal bovine serum (HI-FBS) and 1% antibiotics (penicillin–streptomycin). MNT-1 human melanoma cells were obtained as a kind gift from Dr. Michael Marks, University of Pennsylvania, and were cultured in 18% HI-FBS, 1% minimum essential media (MEM), 1% antibiotics, and 10% AIM-V media (Invitrogen, Carlsbad, CA, USA). Primary human epidermal melanocytes isolated from moderately pigmented neonatal foreskin (HEMn-MP) and lightly pigmented neonatal foreskin (HEMn-LP) were purchased from Cascade Biologics (Portland, OR, USA) and cultured in Medium 254 (Cascade Biologics) supplemented with 1% human melanocyte growth supplement (HMGS) and 1% antibiotics. All the cells were cultured in a 95% humidified atmosphere with 5% CO_2_ in a 37 °C incubator.

### 4.4. MTS Cell Viability Assay

To test DOLE for its effects on melanin synthesis and melanin secretion, we first evaluated DOLE for any cytotoxicity using MTS assay (Promega CellTiter Aqueous One, Madison, WI, USA). MTS is a tetrazolium salt which is reduced to purple-colored formazan upon reaction with mitochondrial dehydrogenases. Briefly, B16F10 cells were seeded at 1 × 10^4^ cells/well in a 96-well plate for 24 h, followed by the replacement of culture medium containing DOLE at various concentrations, and cultures were maintained for 48 h. At this point, the medium was aspirated, replaced by 100 μL of fresh medium containing 20 μL of MTS reagent, and incubated for 40 min. After this step, a total of 100 μL was aliquoted in a new 96-well microplate and the absorbance was read at 490 nm using a Versamax^®^ microplate reader. Cell viability was determined from the absorbance values relative to control groups and expressed in %.

To test DOLE for cytotoxicity to MNT-1 human melanoma cells, 2 × 10^4^ cells/well were dispensed in a 96-well plate for 24 h after which DOLE was added, and the cultures were maintained for a period of 48 h. After this step, MTS assay was conducted similar to the method described above (except an incubation period of 2.5 h with MTS reagent was conducted).

To test DOLE for cytotoxicity to primary melanocytes, HEMn-LP or HEMn-MP (2 × 10^4^ cells/well) were inoculated in a 96-well plate for 24 h after which DOLE at various concentrations was added, and cultures were maintained for a period of 48 h. After this step, MTS assay was conducted similar to the method described above (except an incubation period of 90 min with MTS reagent was conducted), and results were expressed as % normalized to untreated control.

### 4.5. Extracellular and Intracellular Melanin Assay

B16F10 cells (1 × 10^5^ cells/well) were seeded in 12-well plates and incubated for 24 h. At this point, DOLE was added at various concentrations and the cultures were maintained for another 48 h. After the treatments, the culture supernatants were centrifuged and transferred to a 96-well plate, and the absorbance was read at 475 nm using a microplate reader to estimate extracellular melanin. For intracellular melanin quantitation, the cells were detached from the culture plates (by adding TrypLE™ Express Enzyme (1X; Gibco™, Thermo Fisher Scientific Inc., Waltham, MA, USA) and incubating at 37 °C for 5 min), washed in PBS, and observed for changes in color of cell pellets. At this point, 250 μL of 1N NaOH was added to cell pellets and heated to 70 °C to solubilize melanin. The absorbance of lysates was read at 475 nm and a portion of the lysate was used to evaluate total protein content using bicinchoninic acid (BCA) assay. The extracellular and intracellular melanin absorbances were normalized to total protein content and expressed as relative melanin levels of untreated control in %.

To test DOLE in MNT-1 cells, 2.2 × 10^5^ cells/well were cultured in 12-well plates for 24 h, after which DOLE was added and cells were maintained for a duration of 48 ours. At this point, the medium was assayed for extracellular melanin, while the cells were processed for intracellular melanin estimation, similar to the method described above, and results were normalized to total protein content and expressed as % of control.

To test DOLE for melanin levels in primary melanocytes, HEMn-LP (2.3 × 10^5^ cells/well) or HEMn-MP (1.5 × 10^5^ cells/well) were cultured for 24 h in 12-well plates, followed by treatment with DOLE at various concentrations, and the cultures were maintained for 48 h. At this point, the culture medium was collected, centrifuged, and aliquoted in a 96-well plate to estimate extracellular melanin. The cells were processed for the estimation of intracellular melanin similar to the method described above. The extracellular and intracellular melanin absorbances were normalized to total protein content and expressed as relative melanin levels as a % of untreated control. 

### 4.6. Cellular Tyrosinase Activity

B16F10 cells were plated in 24-well plates at a density of 5 × 10^4^ cells/well for 24 h, followed by addition of DOLE at various doses, and further incubated for 48 h. At the end of treatments, cells were harvested, washed in PBS, and lysed. After centrifugation (6000× rpm for 5 min at 4 °C), 50 µL of lysates was aliquoted in a 96-well microplate and 150 µL of 3 mM solution of L-DOPA substrate solution (freshly prepared in 50 mM phosphate buffer, pH 6.8) was added. The absorbance was then measured at 475 nm in kinetic mode for 30 min (every 30 s) using a microplate reader. The % tyrosinase activity was calculated from the slope of the linear range of the velocities of inhibition and was normalized to the total protein content.

MNT-1 cells (1.5 × 10^5^ cells/well) were plated in 12-well culture plates for 72 h, followed by the addition of DOLE, and cultures were maintained for another 48 h. At this point, cells were processed for determination of tyrosinase activity similar to the method described above.

HEMn-MP cells (1.1 × 10^5^ cells/well) were plated in 12-well culture plates for 24 h, followed by the addition of DOLE, and cultures were maintained for another 48 h. In the case of HEMn-LP cells, 2.3 × 10^5^ cells/well were plated in 12-well culture plates for 72 h, followed by the addition of DOLE, and cultures were maintained for 48 h. At this point, LP or MP cells were processed for determination of tyrosinase activity. Briefly, cells were lysed and centrifuged; 25 µL was aliquoted in a 96-well plate with the addition of 75 µL of 3 mM L-DOPA solution, and the absorbance was measured every 30 s for a period of 30 min using a microplate reader. The % tyrosinase activity was calculated similar to aforementioned method.

### 4.7. Quantitation of Dendricity in Human Melanocytes

MP cells (0.25 × 10^5^ cells/well) were cultured in 12-well plates for 72 h and then treated with DOLE for 48 h. LP cells (2.3 × 10^5^ cells/well) were cultured in 12-well plates for 24 h and then treated with DOLE for 48 h. At the end of treatments, the morphology of LP or MP cells was analyzed using a Nikon Labphot microscope with a digital camera and the computer-interfaced NIS Elements 5.0 imaging software package. Dendrite lengths were measured from the center of each cell to the end of the cytosolic projection. Total dendrite length (TDL) was calculated by the addition of individual dendrite lengths of each cell which were digitally traced using the NIS software. Numbers of dendrites per cell in untreated and DOLE-treated groups were manually counted from images, while the average dendrite length (ADL) was calculated as the ratio of TDL and number of dendrites. For the quantitation of dendricity, a total of up to 50 cells were analyzed in experiments on HEMn-LP cells and up to 70 cells were analyzed in experiments on HEMn-MP cells.

### 4.8. Melanin Assay in Cocultures of Human Melanocytes

The method for coculture assay was based on the method reported in our previous study [49] that is similar to that reported in another study [50]. Briefly, 1.35 × 10^5^ LP or MP cells were plated in a 6-well plate in complete medium for 24 h. After that, 2.7 × 10^5^ HaCaT cells were added to melanocyte cultures in serum-free keratinocyte growth medium (SF-KGM, Gibco) and the cocultures were continued for 24 h. At this point, DOLE was added and the cocultures were maintained in SF-KGM medium for a duration of 48 h, followed by the estimation of extracellular and intracellular melanin levels based on the method described earlier.

### 4.9. Statistical Analysis

One-way analysis of variance (ANOVA) with Dunnett’s post hoc test was used for comparison of multiple groups, while an unpaired Student’s *t*-test was used for comparing two groups. All statistical analyses were run using GraphPad Prism software (version 8.0, San Diego, CA, USA), and differences were considered statistically significant at *p* < 0.05. All data are reported as mean ± standard error of mean (SEM).

## 5. Conclusions

In summary, our results demonstrate a proof of principle for DOLE as a pro-melanogenic agent for the treatment of hypopigmentation disorders of the skin. The pro-melanogenic capacity of DOLE incorporates an exclusive stimulation of the dendrite length in human melanocytes from Caucasian and Asian skin, which points to a unique mechanism of action for this plant extract not reported before. Future studies to elucidate the effects of DOLE on melanosome export and the molecular mechanisms related to the effects on dendricity, as well as evaluation of the pro-melanogenic capacity of DOLE in a skin tissue model, are warranted.

## 6. Patents

Frutarom owns the technology and patents for the composition of standardized dry olive leaf extract (DOLE; Benolea^®^).

## Figures and Tables

**Table 1:** Dendricity parameters in HEMn-LP cells after treatment with DOLE at 200 μg/mL for 48 h.

Treatment	Number of Dendrites	Total Dendrite Length (μm)	Average Dendrite Length (μm)
Control	2.8 ± 0.12	271.61 ± 12.76	97.84 ± 2.64
DOLE	3.09 ± 0.18	355.75 ± 24.91 ^#^	115.6 ± 3.23

All values are mean ± SEM; ^#^ denotes *p* < 0.01 vs. control; unpaired Student’s *t*-test.

**Table 2:** Dendricity parameters in HEMn-MP cells after treatment with DOLE at 200 μg/mL for 48 h.

Treatment	Number of Dendrites	Total Dendrite Length (μm)	Average Dendrite Length (μm)
Control	3.10 ± 0.13	325.31 ± 15.11	106.73 ± 2.94
DOLE	4.0 ± 0.27 *	561.88 ± 46.30 *	137.73 ± 4.68 ^#^

All values are mean ± SEM; ^#^ denotes *p* < 0.01 and * denotes *p* < 0.0001 vs. control; unpaired Student’s *t*-test.

## Data Availability

Not applicable.

## Supplementary Materials

The following are available online at https://www.mdpi.com/1424-8247/14/3/252/s1, Figure S1: (A) Human keratinocyte (HaCaT) cell viability and (B) normal human dermal fibroblast (NHDF) cell viability in the presence of different concentrations of DOLE, after 48 h. Treatment evaluated using MTS assay. Data for (A) are mean ± SEM of at least two independent experiments. Data for (B) are mean ± SEM of triplicates. Figure S2: Viability of human melanocytes from (A) LP and (B) MP in the presence of different concentrations of DOLE, after 48 h of treatment, evaluated using MTS assay. All data are mean ± SEM of duplicates.
