# Supplementary material for: A Novel Pro-Melanogenic Effect of Standardized Dry Olive Leaf Extract on Primary Human Melanocytes from Lightly Pigmented and Moderately Pigmented Skin
Source: Pharmaceuticals (Basel). 2021 Mar 11;14(3):252. doi: 10.3390/ph14030252 (PMC7999707; doi:10.3390/ph14030252)
Supplement: Supplementary file 1 [file pharmaceuticals-14-00252-s001.zip › Supplementary Information dole.docx]

**Supplementary Information**

A Novel Pro-Melanogenic Effect of Standardized Dry Olive Leaf Extract on Primary Human Melanocytes from Lightly-Pigmented and Moderately-Pigmented Skin

Shilpi Goenka^1^, *, and Sanford R. Simon^1,2,3^

^1^Department of Biomedical Engineering, Stony Brook, NY, USA

^2^Department of Biochemistry and Cellular Biology, Stony Brook, NY, USA

^3^Department of Pathology, Stony Brook University, Stony Brook, NY, USA

*Correspondence: shilpi.goenka@stonybrook.edu

**Supplementary Methods**

*Cytotoxicity Assay in human keratinocytes and fibroblasts*

Human keratinocytes (HaCaT) were obtained from AddexBio (San Diego, CA) and primary human dermal fibroblasts (NHDF) were purchased from Lonza; both cells were cultured in DMEM with 10% HI-FBS and 1% antibiotics. For testing cytotoxicity of DOLE with keratinocytes, MTS assay was conducted. Briefly 1 × 10^4^ cells/well were plated in 96-wells and compounds added for 48 hours. MTS assay was then conducted, and plate incubated for 1 hour and then 100 μL of supernatants were aliquoted into 96 well plates and absorbance read at 490 nm using microplate reader.

For testing cytotoxicity of DOLE to primary human dermal fibroblasts, 1 × 10^4^ cells/well were seeded in 96-well plates and after 24 hours, DOLE at concentrations of 10-200 μg/mL was added to the wells and incubated for 48 hours. At this point, MTS assay was conducted, and the absorbance was read after one hour of incubation of plates and results expressed as % with control.

**Supplementary Figures**


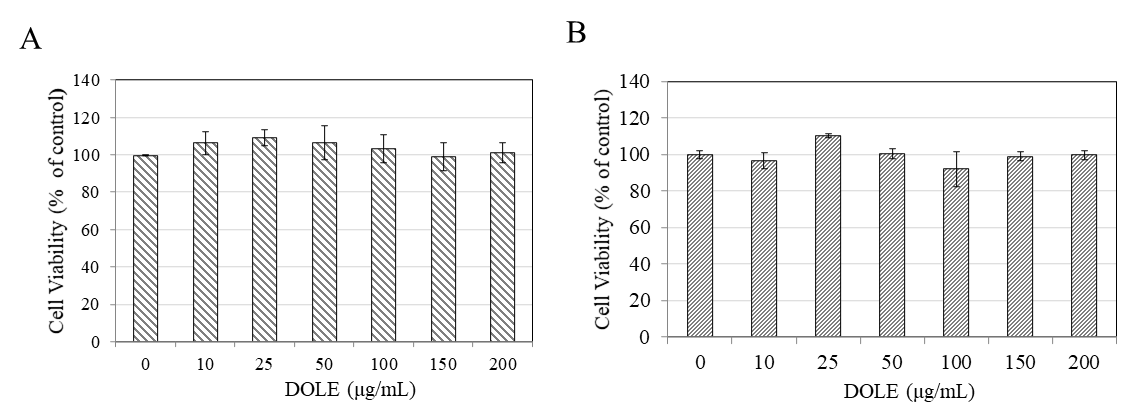


**Figure S1. A)** Human keratinocytes (HaCaT) cell viability and **B)** Normal human dermal fibroblast (NHDF) cell viability in the presence of different concentrations of OLP, after 48 hr. treatment evaluated using MTS assay. Data for A) is mean ± SEM of at least two independent experiments. Data for B) is mean ± SEM of triplicates.


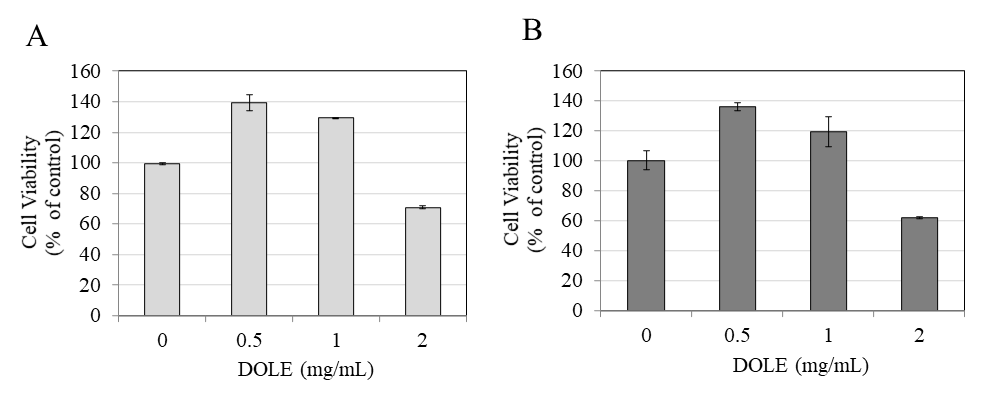


**Figure S2.** Viability of human melanocytes from **A)** LP and **B)** MP in the presence of different concentrations of DOLE, after 48 hr treatment evaluated using MTS assay. All data is mean ± SEM of duplicates.
